# Reviewed article: Lerm BR, Pletsch V, Géa MFF, Santos IS, Regner AP, Giugliani C. Social vulnerability of children and adolescents who are victims of sexual violence assisted in a specialized service: descriptive study, Porto Alegre and metropolitan region, 2018. Epidemiol Serv Saude. 2026:35;e20240780

**DOI:** 10.1590/S2237-96222026v35e20240780.c

**Published:** 2026-10-30

**Authors:** Alberto Madeiro

**Affiliations:** 1 Universidade Federal do Piauí, Teresina, PI, Brazil Universidade Federal do Piauí Teresina PI Brazil

## First round comments

Prezadas Beatriz, Camila, Vanessa, Fernanda, Maria, Iná e Andréa,

O tema da violência sexual contra crianças e adolescentes é muito relevante e o manuscrito tem grande potencial. Entretanto, há necessidade de múltiplos ajustes para tornar possível sua publicação.

Os pareceristas realizaram revisões minuciosas e ofertaram sugestões valiosas. Destaco que as recomendações sinalizadas em detalhes por eles são fundamentais para o aprimoramento de todo o artigo.

Reforço abaixo alguns desses pontos e apresento outros:

1. Concordo que o Resumo necessita de ampla revisão para se adequar ao padrão exigido pela RESS (recomendo reler as “Instruções aos autores”). Pelo menos dois aspectos destacados pelos revisores merecem ênfase. Primeiro, espera-se que os autores ofertem dados numéricos (proporções, medidas de associação ou correlação) dos resultados apresentados. Segundo, as conclusões devem responder aos objetivos e serem baseados nos dados mostrados no próprio resumo.

2. A seção de Introdução é bem estruturada no geral, mas ganharia em robustez se ofertasse um panorama (mesmo que breve) da violência sexual contra crianças e adolescentes no contexto do Rio Grande do Sul, o que ajudaria na justificativa do estudo. Uma busca rápida no Scielo evidenciou a já existência de vários artigos consolidando informações sobre o tema no estado.

3. A leitura do artigo realmente deixa dúvidas se os dados se referem ao ano de 2018 ou 2022. Além disso, na seção de Métodos, solicito que todas as variáveis tenham suas categorias declaradas (exemplo: “faixa etária (em anos: < 1; 1-5; 6-9; 10-14; 15-18”).

4. Sugiro padronizar os nomes das variáveis em todo o manuscrito: “proporção de casos” (exibido na tabela 3) é o mesmo que “taxa de casos” (descrito no texto)? Se sim, uniformizar. Outra questão: os dados sobre “razões” só aparecem no texto (não na tabela 3).

5. Tabela 3: a análise dessa tabela demonstra apenas 4 municípios da região metropolitana, porém na Figura 1 eles parecem ser em maior número. Solicito que os dados de todos os municípios (e também das UDS) sejam mostrados na tabela 3 e não apenas aqueles com maiores taxas.

6. Julgo que a Figura 1 é insatisfatória. Apesar de visualmente informativa no que diz respeito às regiões onde as taxas eram mais ou menos elevadas, não há como identificar os valores numéricos da correlação (como o texto dá a entender). Uma vez que a correlação entre IVS e IDS versus taxas é uma das formas de análise, recomendo que os dados sejam mostrados de forma detalhada em tabela, por exemplo.

7. Rever o tópico “Disponibilidade de dados”: nesta seção deve ser informado onde os leitores podem encontrar o banco analisado na presente pesquisa, se necessário (banco de dados utilizado para análise, bem como códigos e demais dados pertinentes para assegurar transparência na pesquisa).

Favor observar a política editorial da RESS, item Dados abertos: “Os dados de pesquisa (bancos de dados, códigos, métodos e outros materiais utilizados e resultantes da pesquisa) idealmente devem ser depositados em repositórios de dados confiáveis como o repositório SciELO Data (lista disponível em https://wp.scielo.org/wp-content/uploads/Lista-de-Repositorios-Recomendados_pt.pdf), preferencialmente com um identificador persistente como o digital object identifier (DOI). O acesso a tais dados com o link para o repositório deverá ser informado na seção “Disponibilidade de dados”. Deve haver citação dos dados no texto, preferencialmente nos “Métodos”, e a referência completa deve ser incluída na seção de “Referências”, seguindo exemplo:

Andrade M. Estudo de genes em ratos albinos na América Latina. OSF , 2018, ASM0000v1. http://dx.doi.org/10.1590/0123-45620187214”.

Apesar de a RESS estimular fortemente que os dados sejam depositados em repositórios, essa ação ainda não é obrigatória. Se os autores não puderem ou desejarem o depósito, devem declarar algo como “Os dados gerados durante o estudo estão disponíveis com o autor correspondente, mediante pedido”.

8. A seção de Referências realmente necessita ampla revisão. Há títulos em caixa alta, ausência de páginas, links inoperantes... (rever Instruções aos autores).

Recommendation: Major revision

## Second round comments

Parabenizo as autoras pela significativa reformulação do manuscrito, tornando-o mais estruturado.

Solicito, ainda, atenção para os tópicos listados abaixo:

1. Título: inserir o ano em que os dados foram coletados. Também inserir o tipo de estudo.

2. Introdução/segundo parágrafo: rever a grafia da palavra “iniquidade”.

3. Introdução: recomendo padronizar a declaração de objetivo (último parágrafo) com o objetivo descrito no Resumo.

4. Métodos: retirar a inicial maiúscula de “Capital”.

5. Métodos: sugiro declarar quantas são as Unidades de Desenvolvimento Humano avaliadas.

6. Resultados: empregar ponto para separar a unidade de milhar. Exemplo: “1.913”. Rever em todo o documento.

7. Resultados/segundo parágrafo/nível agregado: está com fonte diferente (não é Times New Roman).

8. Tabela 1:

- retirar o espaço dos intervalos das categorias de faixa etária (“1-5”);

- explicar o significado das siglas “IVS”, “IDH” e “RM”;

- “IDH” está com fonte diferente de Times New Roman.

9. Resultados/nível agregado/segundo parágrafo: sugiro que esse parágrafo (com os pontos de corte dos quintis) seja deslocado para a seção de Métodos. Ele não oferta nenhum resultado.

10. Tabela 3: retirar as linhas centrais horizontais e verticais.

11. Referências:

- estão em fonte diferente de Times New Roman;

- excluir a menção à data de acesso e endereço (exceto para documentos eletrônicos). Exemplo: “Rates SMM, Melo EM, Mascarenhas MDM, Malta DC. Violence against children: an analysis of mandatory reporting of violence, Brazil 2011. Cienc Saude Colet. 2015;20:655-65”.

12. Para as tabelas do Material Suplementar: empregar a mesma formatação das tabelas do texto. Explicar as siglas no título (quando possível) ou como nota.

**Recommendation:** Minor revision

## Third round comments

Caras autoras, solicito ainda revisão dos seguintes pontos:

1. Resumo/Resultados: em todo o documento, padronizar os percentuais com 01 casa decimal (exemplo: “vítimas de violência intrafamiliar (53,3%)”).

2. Introdução/último parágrafo:

- recomendo padronizar (deixar igual) a declaração de objetivo com o objetivo do Resumo;

- penso que a última oração acrescentada (“Através da metodologia de geoprocessamento...”) está deslocada aqui, uma vez que ela antecipa passos metodológicos que serão apresentados a seguir. Sugiro excluir.

3. Métodos/Geoprocessamento:

- sugiro apresentar essa subseção após “Variáveis”;

- as duas frases a seguir, apesar de boas, antecipam os resultados. Sugiro reavaliar se elas não ficariam melhor contextualizadas na seção de Resultados:

a. “Do total de 722 unidades de Porto Alegre e Região Metropolitana, 426 (59,0%) apareceram em nossa análise”;

b. “As 296 Unidades de Desenvolvimento Humano que não tiveram nenhum caso atendido foram representadas no mapa (Figura 1) com preenchimento tracejado. Destas, 128 (43,2%) pertencem a Porto Alegre”.

4. Referência 7 e 18: faltam as páginas.

Recommendation: Minor revision

## Fourth round comments

1. De fato, o material suplementar também necessita estar dentro das normas para tabelas e/ou gráficos e os aspectos da formatação passaram despercebidos na última revisão. Sugiro as seguintes mudanças:

- dada a grande quantidade de informações (tabelas muito longas), sugiro que o material suplementar seja excluído;

- como ponderam as autoras, o que motivou a oferta de material suplementar foram os dados mostrados na Tabela 4. Nessa tabela, houve decisão de apresentar apenas os resultados dos 5 municípios e 5 UDS com maiores taxas de casos, dificultando para o leitor a comparação com os demais e levando a editoria a solicitar todos os dados. Talvez uma estratégia alternativa seja mostrar também os 5 municípios e 5 UDS com menores taxas ou, ainda, exibir os resultados dos 10 municípios e UDS com maiores taxas. Avaliem;

- “Da mesma forma, elaborou-se um mapa com os valores do IDH, disponibilizado no material suplementar (Figura Suplementar)”. Recomendo avaliar a possibilidade de juntar a Figura 1 + Figura Suplementar (A/B/C/D), de forma a não ter nenhum material suplementar e mostrar os dados do IDH dentro

- “As estimativas de taxa de casos, número de casos, população menor de 18 anos, IVS e IDH de todos os municípios e de todas as Unidades de Desenvolvimento Humano, incluindo as que não tiveram casos atendidos no serviço, estão disponíveis no material suplementar (Tabela Suplementar 1-3)”. Sugiro excluir esse trecho.

2. As siglas “IVS” e “IDH” não são explicadas na primeira vez que aparecem no texto (Métodos). A explicação aparece em parágrafos posteriores.

3. Há discrepância entre o valor do percentual da violência intrafamiliar mostrada no Resumo (“53,9%”) e texto da seção de Resultados (“53,3%”). Qual o correto? Por favor, revisar todos os valores numéricos do Resumo, texto e tabelas.

4. Resultados: sugiro excluir o subtítulo “Nível agregado”.

5. Títulos das tabelas 1 e 2: para atender a padronização da RESS, sugiro, por exemplo, “Frequência (n) e percentual (%) das características sociodemográficas...”.

6. Referências: sugiro padronizar as referências dos artigos de periódicos da seguinte maneira: “Morehouse SI, Tung RS. Statistical evidence for early extinction of reptiles due to the K/T event. J Paleontology. 1993;17(2):198-209”. Excluir as menções à internet, data de publicação, DOI ou endereço eletrônico.

Recommendation: Minor revision

